# Serolological Profile Among HBsAg-Positive Infections in Southeast China: A Community-Based Study

**DOI:** 10.5812/hepatmon.7604

**Published:** 2013-01-23

**Authors:** Ping Chen, Chengbo Yu, Wei Wu, Jinghua Wang, Bing Ruan, Jingjing Ren, Shigui Yang, Kaijin Xu, Liang Yu, Lanjuan Li

**Affiliations:** 1 State Key Laboratory for Diagnosis and Treatment of Infectious Diseases, The First Affiliated Hospital, College of Medicine, Zhejiang University, Hangzhou, China

**Keywords:** China, Hepatitis B, Hepatitis B e Antigens, Residence Characteristics, Serologic Tests

## Abstract

**Background:**

Hepatitis B virus (HBV) infection has remained a significant public health problem. Generating a large-scale, community-based profile of HBV infection in China is essential to prevention of the disease.

**Objectives:**

The current study was designed to investigate HBV-infected individuals at the community level and determine the age distribution, hepatitis B e antigen (HBeAg) positivity and its related risk factors, relationship among serological markers.

**Patients and Methods:**

A cross-sectional, community-based survey was carried out without age restriction, in 12 communities of two counties. The study population was selected by random multistage cluster sampling. Serological samples and demographic information were collected from 8439 HB surface antigen (HBsAg)-positive individuals.

**Results:**

The constituent ratio of individuals with HBsAg-positive infections was lowest among persons aged < 20 years (0.4%) and the highest among persons aged 40-49 years (33.2%). The HBeAg-positive rate among infected individuals was 18.5%, and the constituent ratio decreased with increasing of age. The HBeAg-positive rate in males (21.9%) was significantly higher than in females (14.7%), and was higher among coastland inhabitants (22.9%) than among plains inhabitants (12.9%). Among the 1561 HBeAg-positive individuals, 91.0% were HBV DNA-positive. However, of the 6878 HBeAg-negative individuals, only 45.4% were HBV DNA-positive, and the HBeAg-positive rate was significantly different at different levels of HBV DNA expression. The proportion of detectable HBV DNA levels was significantly higher in individuals with elevated ALT, compared to those with normal ALT, regardless of HBeAg-positivity.

**Conclusions:**

The HBV prevalence remained high in the > 20 age group. The positivity of HBeAg was related to age, region, and sex. Testing HBeAg and serum ALT levels were effective ways to assess HBV infectiousness in community-level hospitals in China.

## 1. Background

The hepatitis B virus (HBV) infection has remained a significant public health problem worldwide, accounting for around one million annual deaths from HBV-associated complications of liver failure, cirrhosis, and hepatocellular carcinoma (HCC) (1, 2). China is classified as a highly endemic region for HBV infection (2, 3). In HBV infection, HBeAg is an important biological indicator of virus replication status, a patient’s infectivity, and extent of ongoing liver injury (4). In the clinic, an HBeAg-positive result indicates early phase or active HBV infection. Therapeutic intervention with anti-viral agents can suppress HBeAg expression to undetectable levels. The possibility of HBeAg seroconversion is 2-15% lower in HBV-infected patients who do not receive anti-viral treatment (5). Unfortunately, the ever-evolving HBV genome has challenged our understanding of this disease. Novel and robust variations in the core (C) gene’s pre-C and C promoter have led to increased rates of HBeAg-negative infection worldwide. In China, the overall HBeAg-positive population has decreased over the past three decades, going from 81.3% in 1980 to 46.3% in 2003 (6). To date, very few large-scale community-based surveys of HBV infected individuals have been carried out in China, and the community positivity of HBeAg and its related risk factors have remained unknown. It is important to understand the profile of HBV infection diagnosed at rural hospitals in order to design preventative programs. The current study conducted a community-based survey, where HBV-infected individuals were screened from a random sample of the entire population of 12 communities to determine the HBV infection rate and pathogenic profile in Northern Zhejiang Province.

## 2. Objectives

The current study was designed to investigate HBV-infected individuals at the community level and determine the age distribution, hepatitis B e antigen (HBeAg) positivity and its related risk factors, relationship among serological markers.

## 3. Patients and Methods

### 3.1. Study Site

This study was carried out in the northern region of Zhejiang Province, which is located in the south of the Yangtze River Delta in southeast China.

### 3.2. Study Population and Sampling Strategy

This study was approved by the Ethics Committee of the First Affiliated Hospital at the Zhejiang University College of Medicine. Field work was conducted from March 2010 to October 2011. All participants provided written informed consent. Individuals who were enrolled in the study had HBsAg-positivity for at least six months and were permanent residents of the northern Zhejiang Province region. The target study population was selected from a list of residents using the random multistage cluster sampling approach. First, two municipalities with different landscapes (plain vs. coastland) were chosen randomly. Next, we selected one county/city (equivalent to county) from each municipality: Shaoxing (plain) and Yuhuan Island (coastal). The towns/sub-districts in each region were divided into three different levels according to economic status, which was defined according to the current year’s gross domestic product (GDP) obtained from the data published by the National Bureau of Statistics (NBS). In each of the three levels, one town/sub-district was randomly selected. Finally, within each town/subdistrict, two villages or residential communities (equivalent to a village) were also selected according to the economical level. The reasearchers conducted HBV-related screening in these 12 village/residential communities to determine all the infected individuals within the entire residential population.

### 3.3. Serological Testing

A 5 mL venous blood sample was collected from each enrolled study participant using strict hygiene and safety guidelines. The patient’s demographic information, including name, sex, age, and district, were recorded. Blood samples were kept in a cold container and immediately delivered to Adicon Clinical Laboratories, Inc. (Shanghai, China) for serum separation and indefinite storage at -30℃. Sample processing and serology was performed by the Central Laboratory (Hangzhou, China). Commercially available enzyme immunoassay kits (Acon Biotech Co., Hangzhou, China) were used to assess hepatitis B virus surface antigen (HBsAg) status. Microparticle enzyme immunoassay kits (Abbott, Chicago, USA) were used to assess HBeAg titer. HBeAg titer > 1 S/CO was considered as HBeAg-positive. The real-time fluorescent PCR system (7300; Applied Biosystems, Inc., Carlsbad, CA, USA) was used to detect HBV DNA levels. DNA value > 103 copies/mL was considered as HBV DNA-positive. Alanine aminotransferase (ALT) was measured to assess liver function. The Architect C8000 automated biochemistry analyzer (Abbott Laboratories, Abbott Park, IL, USA) was used to detect ALT levels. An ALT value > 38 IU/L indicated abnormal function. Verification of test results was carried out towards positive specimens by retesting twice on the same sample using the same kits. Only samples which were positive on both tests were considered reactive.

### 3.4. Statistical Analysis

SPSS software version 17.0 (SPSS, Inc., Chicago, IL, USA) was employed to manage and analyze the data. For categorical variables, the Chi-squared test was used to determine inter-group differences. A P value < 0.05 was considered statistically significant. The 95% confidence intervals (CI) for the proportion of individuals who were Ag-positive, stratified by age and sex, as well as for the whole group, were measured.

## 4. Results

### 4.1. Characteristics of the Study Group

A total of 8875 individuals were recruited, and their serum samples were re-tested. Seventy-five individuals were HBsAg-negative and were excluded from further analysis. An additional 361 individuals lacked corresponding information on donor age, sex or region, and were thus excluded from further analysis. Therefore, a total of 8439 individuals were analyzed, of which 4435 were male and 4004 were female. Shaoxing residents accounted for 3715 of the individuals, and Yuhuan residents accounted for 4724. The age and sex proportions between the two regions were similar. Demographic characteristics of the study population are shown in Table 1.

**Table 1:** Demographic Data of Study Population

County/City	Village/Residential Communities	Population	Participants Interviewed	No. of Valid	Males, %	Age, y, Mean ± SD
**Shaoxing**	Rongshan	9013	579	543	46.8	51.14 ± 12.42
** **	Fusheng	14523	886	846	50.5	49.92 ± 12.54
** **	Pingshui	11642	783	749	46.7	50.11 ± 2.03
** **	Qingfeng	9986	679	654	50.5	48.87 ± 12.61
** **	Fuquan	8432	538	511	45.8	52.62 ± 12.54
** **	Wuyang	6993	451	412	50.0	50.35 ± 13.12
**Yuhuan**	Cangkeng	7812	867	834	54.4	45.42 ± 12.45
** **	Yindong	6021	662	629	57.1	44.89 ± 11.82
** **	Shuanglong	9254	1091	1034	53.9	47.85 ± 12.11
** **	Nanmen	9377	1040	997	57.8	44.36 ± 11.57
** **	Houjiao	7094	812	768	56.3	46.82 ± 12.49
** **	Putian	3926	487	462	55.4	46.21 ± 12.08
**Total**		104073	8875	8439	52.6	47.90 ± 12.51

### 4.2. Distribution of HBV Infection by Age, Sex, and Region

**Figure 1:**
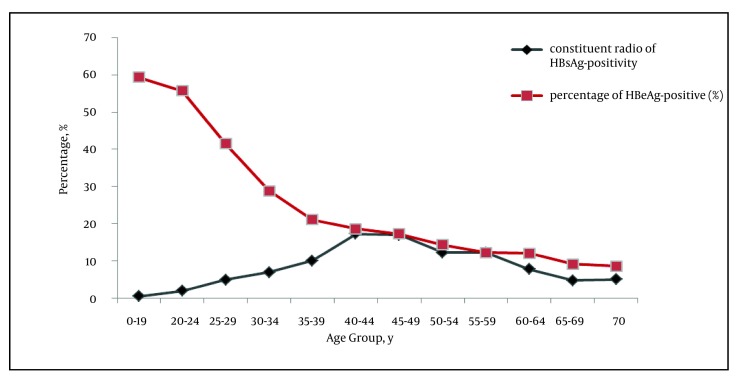
Proportion of Hepatitis B Virus Markers, Stratified by Age Abbreviations: HBeAg, hepatitis B e antigen; HBsAg, hepatitis B surface antigen

**Figure 2:**
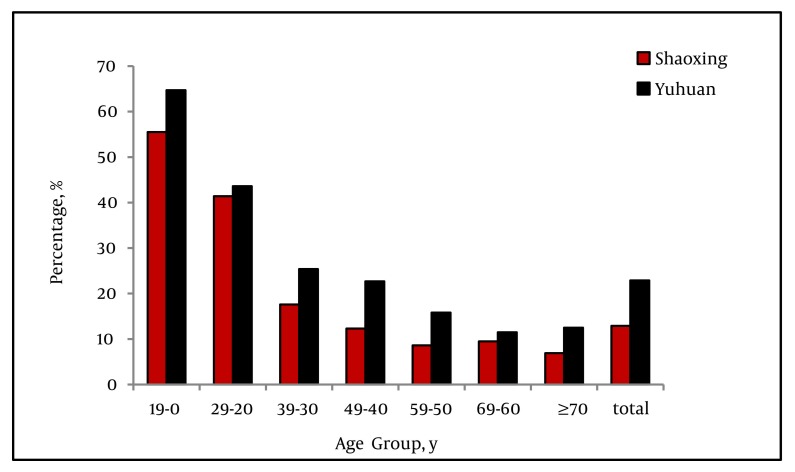
Region-Specific HBeAg Positivityin the HBsAg-Positive Population Abbreviations: HBeAg, hepatitis B e antigen; HBsAg, hepatitis B surface antigen

Of the 8439 HBsAg-positive individuals, the constituent ratio of persons aged < 20 years was the lowest, with a crude rate of 0.4%. The constituent ratio of persons aged 40-49 years was the highest, with a crude rate of 33.2%. There was a peak of HBsAg-positive infection in the fourth decade of life, which declined thereafter (Figure 1). A total of 1561 tested individuals were positive for HBeAg. The positivity of HBeAg in the HBsAg-positive population accounted for 18.5% (95% CI: 17.7%-19.3%). The HBeAg-positive rate was the highest among HBV-infected individuals aged < 20 years (60%), and decreased with increasing age thereafter (P < 0.001), falling to 9.2% in the > 70 years old group (Figure 1). HBeAg positivity among male subjects (21.9%; 95% CI: 20.7%-23.1%) was significantly higher (P < 0.001) than among female subjects (14.7%; 95% CI: 13.6%-15.8%) (Table 2). The HBeAg-positive rate in the coastal Yuhuan area (22.9%; 95% CI: 21.7%-24.1%) was significantly higher than that of plain Shaoxing area (12.9%; 95% CI: 11.8%-14.0%). This significant difference was found for all age groups (Figure 2).

**Table 2:** Sex-Specific HBeAg-Positivityin the HBsAg-Positive Population

	Study Participants, No.	HBeAg-Positive, No.	HBeAg-Positivity, %
**Male**	4435	972	21.9
**Female**	4004	589	14.7 a
**Total**	8439	1561	18.5

Abbreviations: HBsAg, hepatitis B surface antigen; HBeAg, hepatitis B e antigen

^a^P < 0.001vs. Males

### 4.3. Correlation Between HBeAg and HBV DNA

The 1561 HBeAg-positive individuals accounted for 18.5% of the total study population. Among this subset, 91% were HBV DNA-positive. However, of the 6878 HBeAg-negative individuals, 45.4% were HBV DNA-positive. There was a significant difference in HBV DNA positivity between the two groups (P < 0.001). Moreover, the HBeAg-positive rate was significantly different at different levels of HBV DNA expression. The HBeAg-positive rate was only 3.6% in the group with HBV DNA negativity, and it increased in parallel with the increases in HBV DNA copies, reaching 94% in the group with HBV DNA > 107 copies. The mean age of the group with HBV DNA copies > 107 was significantly lower than that of the other groups (P < 0.001) (Table 3).

**Table 3:** Correlation Between HBeAg and HBV-DNA

HBV DNA, Copies/mL	No.	HBeAg		
		**Mean ± SD **	**Positive, %**	**Negative, %**
**< 10^3^**	3898	48.44 ± 12.41	140, 3.6	3758, 96.4
**~ 10^3^**	2705	49.05 ± 11.77	163, 6.0	2542, 94.0
**~ 10^5^**	884	49.85 ± 11.43	363, 41.1	521, 58.9
**~ 10^7^**	952	40.64 ± 13.45	895, 94.0	57, 6.0

Abbreviations: HBeAg, hepatitis B e antigen; HBV DNA, hepatitis B virus DNA

### 4.4. Correlation Among HBeAg, ALT, and HBV DNA

The proportion of detectable HBV DNA levels was slightly but significantly higher in the HBeAg-positive group with elevated ALT (95.4%) compared to those of the HBeAg-positive group with normal ALT (86.4%) (P < 0.001). The proportion of detectable HBV DNA levels was also significantly higher in the HBeAg-negative group with elevated ALT (59.6%) compared to those of the HBeAg-negative group with normal ALT (41.5%) (P < 0.001) (Table 4).

**Table 4:** Correlation Among HBeAg, ALT, and HBV-DNA

	HBeAg-Positive		HBeAg-Negative	
**Serum ALT, %**	< 38 IU/L	> 38 IU/L	< 38 IU/L	> 38 IU/L
**HBV. DNA> 10^3^copies/mL**	656 (86.4)	765 (95.4) a	2245 (41.5)	875 (59.6) a
**HBV DNA < 10^3^copies/mL**	103 (13.6)	37 (4.6)	3164 (58.5)	594 (40.4)
**Total**	759	802	5409	1469

Abbreviations: ALT, alanine aminotransferase; HBeAg, hepatitis B e antigen; HBV-DNA, hepatitis B virus DNA

^a^P < 0.001 vs. normal ALT group

## 5. Discussion

This study quantitatively assessed the burden of all HBV infections from 12 communities in two counties of southeast China. The data from this group provided a recent, community-based serological profile baseline estimate of the success of previous government health programs. In the Zhejiang Province, the EPI vaccination program is considered a success. Since the initiation of the program in 1992, high-risk hepatitis B designation has moved from infants and children (0-9 years old) and adults (30-39 years old) to middle-aged adults (40-49 years old) (7, 8). In the current study, the constituent ratio was the highest among 40-49 year-olds and the lowest among < 20 year-olds, further confirming the former epidemiological discovery. Thus, the current study findings indicate that government programs should continue to target rural population age group 40-49 years-old for HBV-related education and screening. HBV-infected individuals who are diagnosed earlier and receive more timely treatment to decrease secondary complications from CHB and reduce HBV spread. HBeAg-positive patients have shown a better response to anti-viral drugs during the early or active disease stages (9, 10). Moreover, HBeAg-positive infections are considered to be at higher risk for HCC development (11). In our study, infections with HBeAg-positivity only accounted for 18.5% of all participants, which was significantly lower than those reported in previous years in China (12, 13). The increase of HBeAg-negative infections may be related to several factors, including the EPI vaccination program in China, mutations in the pre C region of the virus (14), and/or improved sensitivity of the detection method. In the previous epidemiological studies of French and Italian HBV infections, HBeAg positivity was found to be closely correlated with age (15, 16). However, further analysis was limited by the small sample sizes. In another long-term (12-year) study of 1537 CHB Native American patients, the proportion of HBeAg-positive infections was found to decrease with age (17). In the current study, we found a significant pattern in HBeAg positivity in groups of different ages (P < 0.001). Moreover, HBeAg positivity decreased with age, which is consistent with the former conclusion in the Native American population. It is notable that the patients in the current research were a community-based sample, while those in the other studies were hospital-based samples. The HBeAg positivity among the current study population was 60% in the < 20 years old group, which reflects the immature immune system and immune tolerant phase of children and teenagers. HBeAg positivity among the population aged > 30 years significantly decreased. It is possible that the elderly population may have been infected for many years and have achieved a certain immune clearance mechanism for HBV (4). HBeAg positivity was found to be significantly associated with sex (males: 21.9% vs. females: 14.7%; P < 0.01) in our study, which agreed with previous observations (18). This finding may reflect pathogenesis-related mechanisms involving the sex hormones. However, it is likely that the findings are related to differences in lifestyle or behaviors among males and females, such as smoking, drinking, extensive social range, physical activity, dining out, hygiene, and sexual activity. Luo et al. and Tsai et al. reported that inhabitants of the plain regions had lower rates of infection than those living in island regions (7, 19). However, differences in HBeAg positivity among regions have remained unknown. The current study results indicated that HBeAg positivity had significant regional differences between Shaoxing (plain region) and Yuhuan (island region) (12.9% vs. 22.9%, P < 0.001). It is possible that the different lifestyles practiced in the two regions may be related to the differences in HBeAg positivity. Most people in Yuhuan make a living by fishing. As such, they often live and work in close quarters at sea, sharing food, drink, and daily materials with the bounded community. These practices may increase the severity of hepatitis B, as well as HBV viral replication and infection. However, future research is needed to confirm or deny this hypothesis. HBV DNA is a quantitative serological marker that indicates the replication of HBV. As shown by other studies (20), HBV DNA level has a certain degree of positive correlation with HBeAg level. The current study demonstrated that HBeAg positivity increases with a rise in HBV DNA level. In addition, HBV DNA positivity in the HBeAg-positive group was also significantly higher than that of the HBeAg-negative group (91% vs. 45.4%; P < 0.001). These results suggest the reliability of HBV DNA and HBeAg in indicating virus replication levels. However, few reports have shown the difference between these two markers for their ability to indicate virus replication level. Among the 6878 HBeAg-negative infections, 3120 were HBV DNA-positive (45.4%). In contrast, among the 3898 HBV DNA-negative infections, only 140 were HBeAg-positive (3.6%). These results indicate that HBV DNA testing is likely to be more sensitive than serum HBeAg for HBV diagnosis and assessment of viral replication. It is possible that the HBV copy number remained high in the HBeAg-negative group because these individuals were in an unrecognized disease phase, or that the HBeAg titer had simply fallen below the threshold of detection but not below the threshold of functional impact. Moreover, the HBV pre-C gene mutation has shown to suppress HBeAg expression, while having no effect on HBV DNA replication (16). Although the serological test for HBeAg can basically indicate that HBV is replicating, it is not sufficient to assess HBV replication. Thus, HBeAg levels alone should not be used to design antiviral treatment. The current study also observed the HBV DNA in circulation correlated with ALT levels and HBeAg status. Two studies from Bangladesh suggested that HBV DNA replication should be assessed by traditional serological results and considered together with serum ALT levels to determine HBV status and prognosis(21, 22). In the current study, 95.4% of HBeAg-positive infections with elevated ALT had detectable circulating levels of HBV DNA. However, only 86.4% of HBeAg-positive infections with normal ALT had detectable HBV DNA. Based on these results, it was speculated that serum ALT might be a useful marker of HBV activity and infectiousness in the HBeAg-positive group. Thus, HBeAg, HBV DNA, and ALT could jointly contribute to the comprehensive evaluation of viral replication and host reaction. In most primary hospitals in China, HBV DNA detection is still only performed on a limited basis, due to prohibitively expensive cost. In contrast, detection of HBeAg and ALT is relatively simple and affordable. Therefore, when PCR is not feasible or available to carry out HBV DNA quantitative detection, assessing HBeAg together with serum ALT levels may be sufficient to describe HBV activity and infectiousness. The current study had several limitations which should be considered when interpreting the results. First, we neither detect the site of HBV genome mutations nor did we record the antiviral treatment history among the HBeAg-negative group, and therefore cannot determine the proportion of HBV-infected individuals with the various mutations. Second, the potential correlation of HBeAg, HBV DNA, and ALT in the study population still needs further research. Third, we did not analyze the clinical features of hepatitis, including jaundice, fatigue, and hepatic failure, in the community-based population under study, which may be significant among individuals. In conclusion, it was found that although vaccination against hepatitis B infection has become a standard practice in the study region, the constituent ratio of HBV remains high in the population over age 20, especially among the 40–49 year old age group. HBeAg-positivity was related to age, region, and sex. Although the serological test for HBeAg can indicate that HBV is replicating, it is not sufficient to precisely assess HBV replication itself. Testing HBeAg and serum ALT levels is an effective way to assess HBV infectiousness in community-level hospitals in China.
